# Generation of Functional Inhibitory Synapses Incorporating Defined Combinations of GABA(A) or Glycine Receptor Subunits

**DOI:** 10.3389/fnmol.2015.00080

**Published:** 2015-12-23

**Authors:** Christine L. Dixon, Yan Zhang, Joseph W. Lynch

**Affiliations:** ^1^Queensland Brain Institute, University of QueenslandBrisbane, QLD, Australia; ^2^School of Biomedical Sciences, University of QueenslandBrisbane, QLD, Australia

**Keywords:** inhibitory postsynaptic current, IPSC, GABAergic, glycinergic, neuropharmacology, synaptogenesis, electrophysiology

## Abstract

Fast inhibitory neurotransmission in the brain is mediated by wide range of GABA_A_ receptor (GABA_A_R) and glycine receptor (GlyR) isoforms, each with different physiological and pharmacological properties. Because multiple isoforms are expressed simultaneously in most neurons, it is difficult to define the properties of individual isoforms under synaptic stimulation conditions *in vivo*. Although recombinant expression systems permit the expression of individual isoforms in isolation, they require exogenous agonist application which cannot mimic the dynamic neurotransmitter profile characteristic of native synapses. We describe a neuron-HEK293 cell co-culture technique for generating inhibitory synapses incorporating defined combinations of GABA_A_R or GlyR subunits. Primary neuronal cultures, prepared from embryonic rat cerebral cortex or spinal cord, are used to provide presynaptic GABAergic and glycinergic terminals, respectively. When the cultures are mature, HEK293 cells expressing the subunits of interest plus neuroligin 2A are plated onto the neurons, which rapidly form synapses onto HEK293 cells. Patch clamp electrophysiology is then used to analyze the physiological and pharmacological properties of the inhibitory postsynaptic currents mediated by the recombinant receptors. The method is suitable for investigating the kinetic properties or the effects of drugs on inhibitory postsynaptic currents mediated by defined GABA_A_R or GlyR isoforms of interest, the effects of hereditary disease mutations on the formation and function of both types of synapses, and synaptogenesis and synaptic clustering mechanisms. The entire cell preparation procedure takes 2–5 weeks.

## Introduction

The central nervous system is comprised of circuits of interconnected neurons that serve to process specific types of information. These circuits regulate their own output by feedback and feedforward connections. Knowledge of the physiological properties of the inhibitory and excitatory synapses that mediate these connections is crucial for understanding the electrical behavior of circuits and ultimately of brain function. Fast inhibitory neurotransmission in these circuits is mediated by GABA type-A receptor (GABA_A_R) and glycine receptor (GlyR) chloride channels.

GABA_A_Rs exhibit a particularly broad range of heterogeneity. As members of the pentameric ligand-gated ion channel (pLGIC) family, five subunits are required to form a single functional oligomer. There are 19 GABA_A_R genes (α1–6, β1–3, γ1–3, δ, ε, θ, π, and ρ1–3) with the most common synaptic isoform comprising α1, β2, and γ2 subunits in a 2:2:1 stoichiometry. Although many hundreds of other subunit combinations are theoretically possible, it is thought that around one hundred exist naturally in the brain (Olsen and Sieghart, 2009). GlyRs exhibit far less diversity with only four genes (α1–3, β) in humans (Lynch, 2009). They also belong to the pLGIC family and synaptic GlyR isoforms comprise heteromeric assemblies of α and β subunits in a 2:3 or 3:2 stoichiometry (Durisic et al., 2012; Yang et al., 2012).

Each GABA_A_R or GlyR isoform has a unique physiological and pharmacological profile and it is the unique properties of a particular isoform that are important for the appropriate functioning of a particular network. Disruptions to these properties can result in neurological disorders. For example, hereditary mutations that affect the function of GABA_A_Rs or GlyRs can lead to epilepsy (Macdonald et al., 2010) or human hyperekplexia (Bode and Lynch, 2014), respectively. Other disruptive mechanisms are also possible. For example, a post-transcriptional RNA editing mechanism that is upregulated in temporal lobe epilepsy increases the prevalence of α3 GlyRs incorporating the P185L mutation (Meier et al., 2005; Eichler et al., 2008, 2009). Finally, a range of neurological disorders is known to result from aberrant changes to pLGIC phosphorylation status (Talwar and Lynch, 2014). For example, chronic pain sensitization is caused by prostaglandin-induced phosphorylation of α3 GlyRs (Harvey et al., 2004; Zeilhofer, 2005; Lynch and Callister, 2006) and ethanol-induced phosphorylation of the γ2 GABA_A_R subunit contributes to alcoholism (Qi et al., 2007).

Thus, characterizing the physiological and pharmacological properties of defined GABA_A_R and GlyR isoforms under synaptic activation conditions is essential for understanding how neuronal circuits function in health and disease. However, it is difficult to study individual isoforms in their native neuronal environment due to the multitude of other isoforms present, and the difficulty in pharmacologically or genetically isolating the receptor isoform of interest. The neuron-HEK293 cell co-culture protocols we describe here solve this problem by providing a simple, efficient means of generating functional recombinant inhibitory synapses that selectively incorporate the recombinant GABA_A_R or GlyR isoform of interest.

Co-culture approaches have previously been developed to understand the roles of synaptic adhesion molecules (including neurexin and neuroligin) in the formation of glutamatergic or GABAergic synapses (Scheiffele et al., 2000; Biederer et al., 2002; Dean et al., 2003; Graf et al., 2004; Sara et al., 2005; Kim et al., 2006; Dong et al., 2007; Fuchs et al., 2013) or to investigate the impact of disease-causing neuroligin mutations on GABAergic synaptogenesis (Chubykin et al., 2005; Sun et al., 2011). They have also been employed to characterize the functional properties of inhibitory post-synaptic currents (IPSCs), and have revealed kinetic differences among different GABA_A_R isoforms (Wu et al., 2012; Dixon et al., 2014) and GlyR isoforms (Zhang et al., 2014).

The original co-culture protocol, involving postnatal hippocampal neurons and transfected HEK293 cells, was optimized for the immunohistochemical analysis of glutamatergic and GABAergic synapse development (Biederer and Scheiffele, 2007). A more recent protocol outlined an improved procedure for generating recombinant GABAergic synapses between striatal medium spiny GABAergic neurons and transfected HEK293 cells (Brown et al., 2014). However, this was also optimized for monitoring synapse development rather than for recording IPSCs in mature synapses. We have extended the co-culture approach in three ways. First, we describe the first spinal neuron-HEK293 cell co-culture preparation suitable for the efficient generation of recombinant glycinergic synapses. Second, we have simplified the technique for creating GABAergic synapses by using embryonic cortical neurons grown in serum-free media that does not promote the growth of glia (Brewer et al., 1993; Brewer, 1995). We have also have optimized the technique to facilitate the electrophysiological analysis of GABAergic and glycinergic IPSCs.

## Materials and Methods

### Overview

**FIGURE 1:**
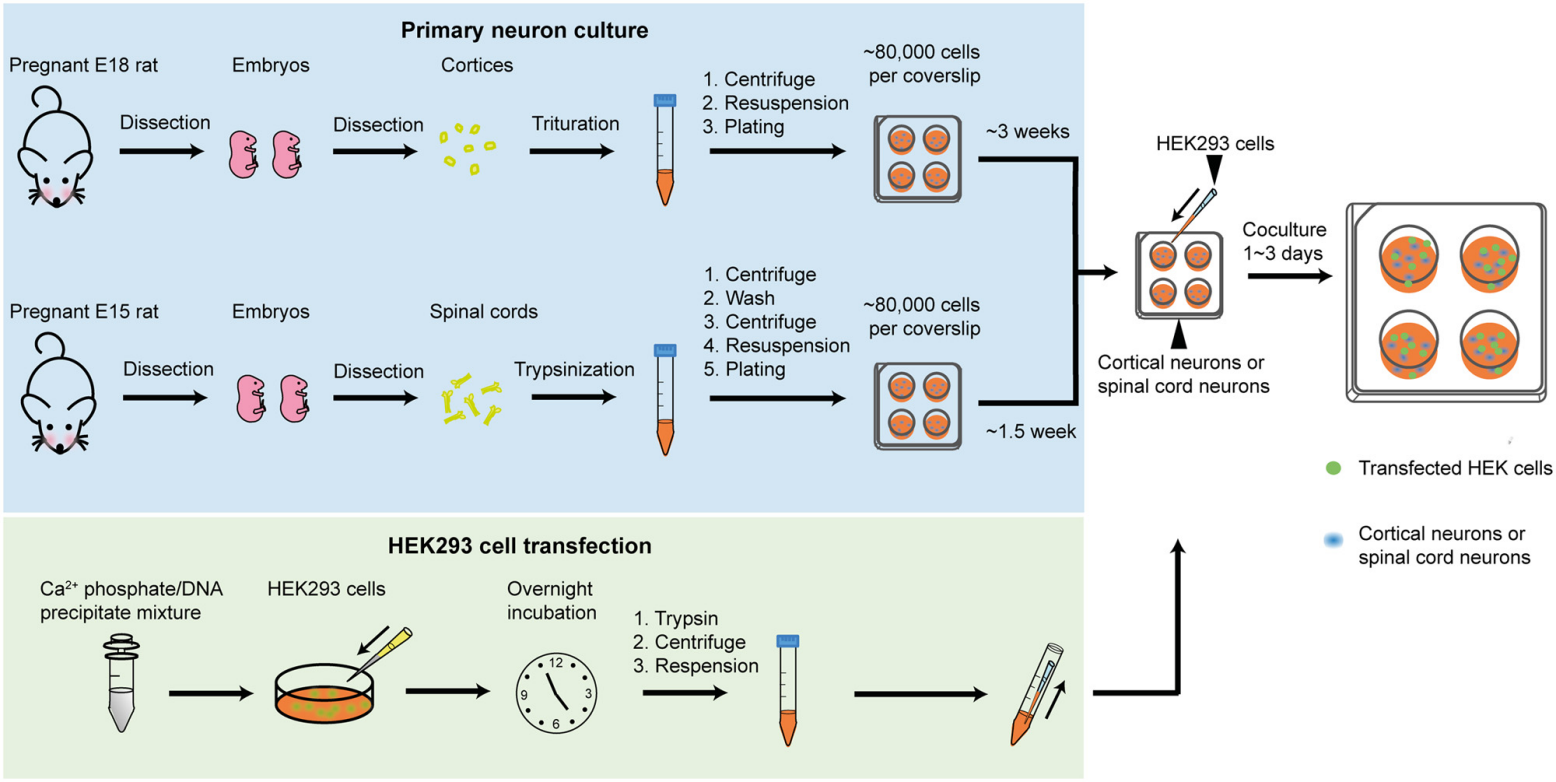
**Overview of the co-culture method**. The blue box describes the steps involved in preparing neuronal cultures (GABAergic in upper row, glycinergic in lower row). The green box describes the steps involved in transfecting HEK293 cells. Inset shows HEK293 cells labeled with a neuroligin-HA antibody (magenta), surrounded by GABAergic neurons forming synapses (as shown by GAD65 labeling in green).

Protocols for all procedures described in this study are detailed in the Supplementary Information. Lists of reagents and equipment are also provided. An overview of the co-culture procedure is presented in **Figure 1**. The cerebral cortex contains large populations of GABAergic interneurons that were used to provide presynaptic GABAergic terminals onto HEK293 cells that recombinantly express the GABA_A_R subunits of interest. Similarly, spinal neurons contain large populations of glycinergic interneurons that were used to provide glycinergic presynaptic terminals onto HEK293 cells expressing GlyR subunits of interest. The main steps in preparing the neuronal cultures are summarized in **Figure 1** (blue box). The steps involved in HEK293 cell transfection are also described in **Figure 1** (green box). An image of a GABAergic neuron forming synaptic contacts with HEK293 cell is shown in the inset.

### Preparation of Neuron Cultures

Euthanasia of timed-pregnant rats was performed via CO_2_ inhalation, in accordance with procedures approved by the University of Queensland Animal Ethics Committee. To produce GABAergic interneuron cultures, E18 rat embryos were surgically removed from timed-pregnant rats and placed into chilled Ca-Mg-free Hank’s Balanced Salt Solution (CMF-HBSS) under sterile conditions. The cortical neuronal tissue was then pinched off using fine forceps, taking care to peel away the meninges to keep glial cell numbers down. The dissected neurons were then triturated, centrifuged and resuspended in Dulbecco’s Modified Eagles Medium supplemented with 10% fetal bovine serum (DMEM-FBS). To produce glycinergic interneuron cultures, E15 rat embryos were surgically removed and placed into ice cold CMF-HBSS under sterile conditions. The spinal cords were then removed and pinned at the wider proximal end while meninges were carefully detached. The dissected neurons were then triturated, centrifuged and resuspended in DMEM-FBS.

In both cases, the cells were then counted and between 40,000 and 80,000 neurons were plated onto each 12 mm poly-D-lysine-coated coverslip in four-well plates. As previously noted, neuronal density is a key consideration: if it is too low it impairs neuronal survival and if it is too high it encourages neuron clumping (Fuchs et al., 2013). Neuronal cultures were always maintained in a 5% CO_2_ incubator at 37°C. After 24 h the entire DMEM-FBS medium was replaced with Neurobasal medium including 2% B27 and 1% GlutaMAX supplements. A second (and final) feed 1 week later replaced half of this medium. In contrast to a previous protocol (Fuchs et al., 2013), we found that antibiotics were unnecessary. Neurons were used in co-culture experiments between 3 and 6 weeks later (for GABAergic co-cultures) or 1–4 weeks later (for glycinergic co-cultures).

### Culture and Transfection of HEK293 Cells

HEK293 cells were cultured in T75 flasks in DMEM-FBS and maintained in a 5% CO_2_ incubator at 37°C. The cells were passaged weekly. Prior to transfection, they were trypsinized and plated onto 35 mm culture dishes at a density of 5000 cells/dish. Following overnight incubation, the cells were transfected via a calcium phosphate co-precipitation protocol, using a total of 0.5–2.5 μg DNA per dish. Then, following incubation for 5–20 h in a 3% CO_2_ incubator, the transfection was terminated by washing twice with divalent cation-free phosphate buffered saline. The cells were then trypsinized, centrifuged, and resuspended in Neurobasal medium (including 2% B27 and 1% GlutaMAX supplements) then seeded onto the neurons. One 35 mm dish of HEK293 cells was sufficient to seed four coverslips of neurons. Once seeded with HEK293 cells, the co-cultures were returned to the incubator overnight to allow synapses to form. Cultures were used for patch clamp recording over the following 2–3 days.

### Plasmid DNA

A total of 0.5–2.5 μg plasmid DNA should be added to each 35 mm dish of HEK293 cells. This amount may vary according to the individual plasmid expression efficiency, the number and ratios of plasmids to be transfected and the transfection method. When using a calcium phosphate co-precipitation protocol, our recommendations are as follows. When expressing synaptic GlyRs, the total plasmid DNA should comprise: 0.2 μg neuroligin 2A, 0.2 μg gephyrin, 0.1 μg EGFP, with the remainder comprising GlyR subunit DNA that varies according to the number of subunits and the ratio of subunit DNA required. For example, when transfecting GlyR α subunits as homomers, add 0.5 μg DNA. When transfecting α and β GlyR subunits in 1:10 or 1:50 ratios add a total of 2 μg DNA. We recommend transfecting α1:β, α2:β, and α3:β subunits in 1:50, 1:50, and 1:10 ratios, respectively (Zhang et al., 2014). When expressing α1β2γ2 GABA_A_Rs, the α1, β2, γ2, EGFP and neuroligin 2A plasmid DNAs should be transfected in a 1:1:4:1:1 ratio with a combined total of 0.5 μg DNA.

**FIGURE 2:**
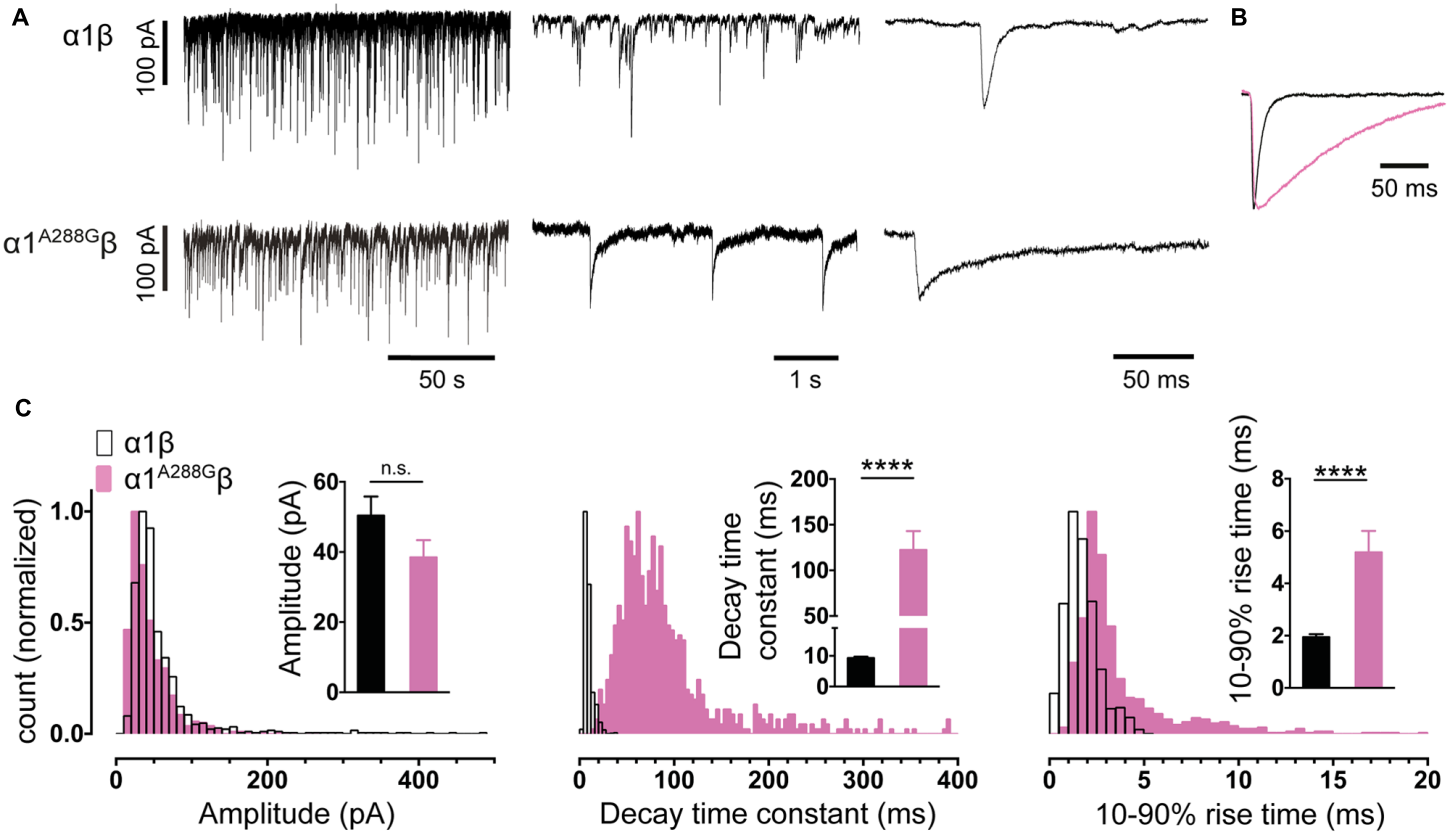
**Comparison of the properties of spontaneous glycinergic inhibitory post-synaptic currents (IPSCs) mediated by α1β GlyRs (in black) and α1^A288G^β GlyRs (in pink). (A)** Sample voltage-clamp recordings at three different time scales for both isoforms. **(B)** Mean IPSC waveforms, each averaged from >200 events from a single cell. **(C)** Mean values and distribution histograms for the peak amplitudes, 10–90% rise times and decay time constants of IPSCs recorded from α1β and α1^A288G^β GlyRs. n.s. = not significant, ^∗∗∗∗^*p* < 0.0001 relative to α1β GlyRs.

**FIGURE 3:**
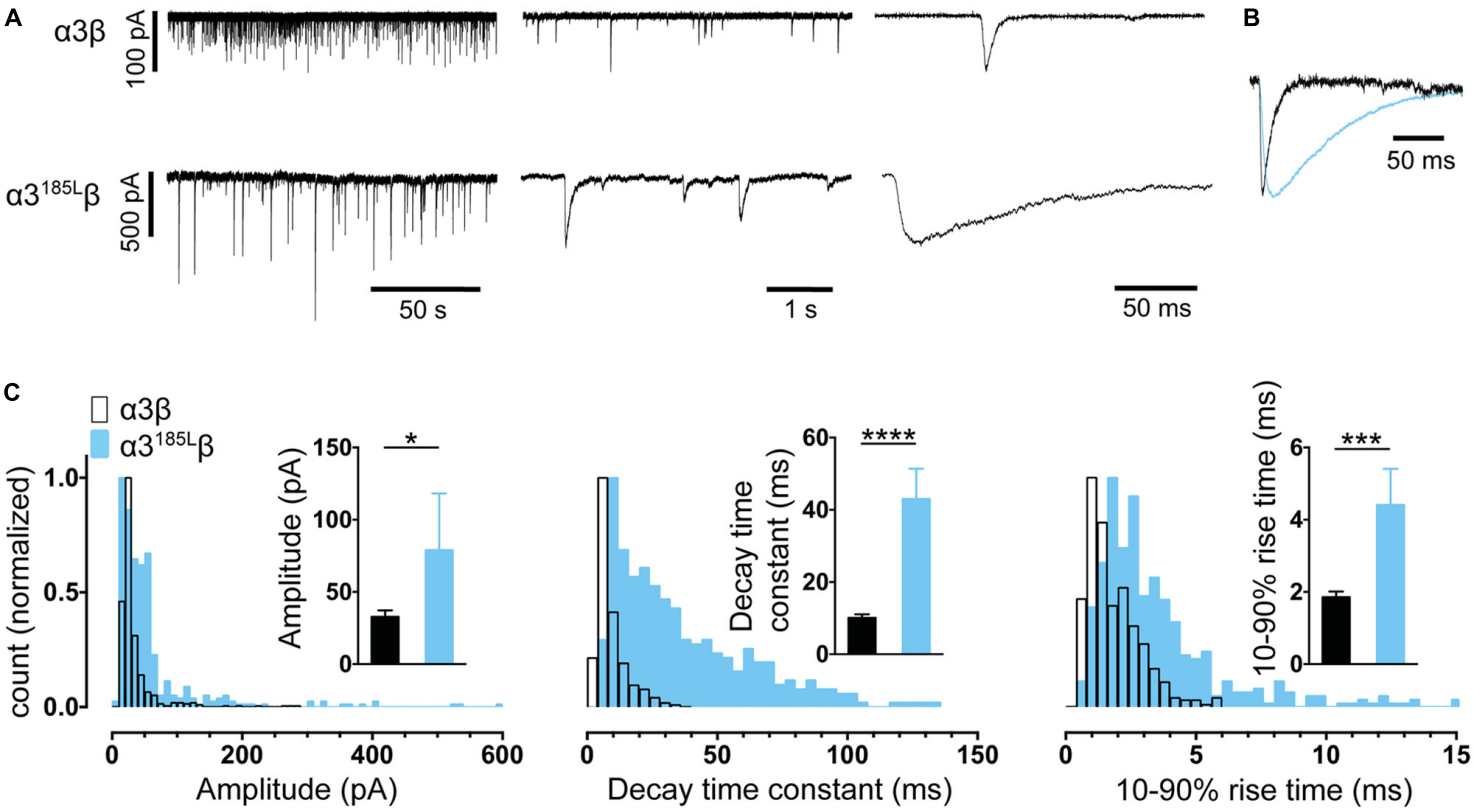
**Comparison of the properties of spontaneous glycinergic IPSCs mediated by α3β GlyRs (in black) and α3^P185L^β GlyRs (in blue). (A)** Sample voltage-clamp recordings at three different time scales for both isoforms. **(B)** Mean IPSC waveforms, each averaged from >200 events from a single cell. **(C)** Mean values and distribution histograms for the peak amplitudes, 10–90% rise times and decay time constants of IPSCs recorded from α1β and α3^P185L^β GlyRs. ^∗^*p* < 0.05, ^∗∗∗^*p* < 0.001, and ^∗∗∗∗^*p* < 0.0001 relative to α3β GlyRs.

**FIGURE 4:**
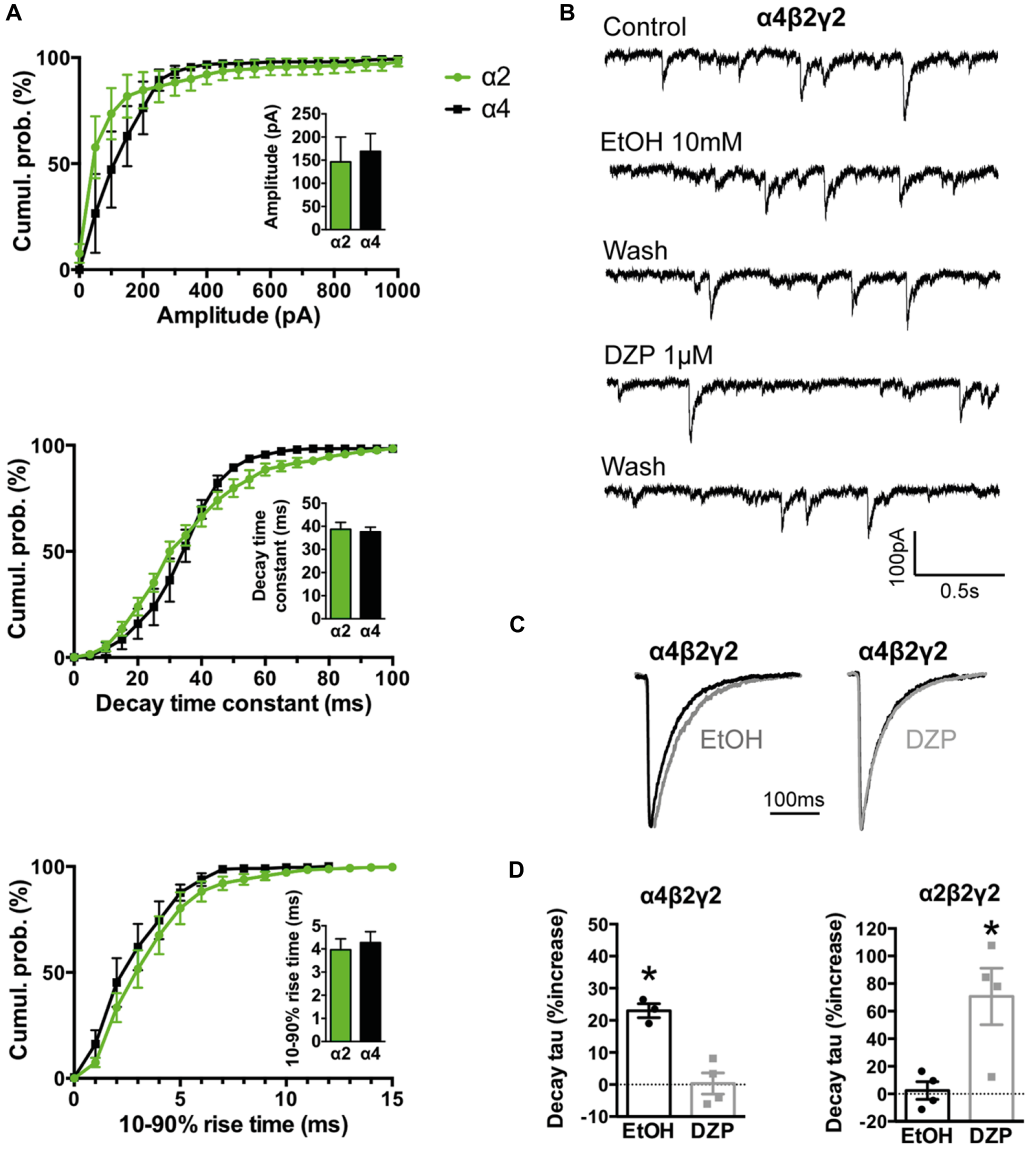
**Comparison of kinetics and pharmacological properties of IPSCs mediated by α2β2γ2 and α4β2γ2 GABA_A_Rs. (A)** Cumulative probability data averaged from four cells expressing α4β2γ2 GABA_A_Rs were compared to previously published data for α2β2γ2 GABA_A_Rs (*n* = 7; Dixon et al., 2014). We found no significant differences in IPSC amplitudes, 10–90% rise times or decay time constants. **(B)** Sample recordings of spontaneous IPSCs mediated by α4β2γ2 GABA_A_Rs before and after the application of 10 mM ethanol and 1 μM diazepam. **(C)** Examples of mean IPSC waveforms mediated by α4β2γ2 GABA_A_Rs, each averaged from >100 events from a single cell, before and after the application of 10 mM ethanol or 1 μM diazepam. **(D)** The decay time constants of IPSCs mediated by α4β2γ2 GABA_A_Rs were significantly prolonged by 10 mM ethanol but not by 1 μM diazepam (left). In contrast, IPSCs mediated by α2β2γ2 GABA_A_Rs were significantly prolonged by 1 μM diazepam but not by 30 mM ethanol (right). Diazepam data for α2β2γ2 GABA_A_Rs were reproduced from (Dixon et al., 2014). ^∗^*p* < 0.05 relative to drug-free control in same cell.

In experiments described in **Figures 2** and **3** we employed plasmid DNAs encoding the human α1 (pCIS), rat α3L (pcDNA3.1), and human β (pcDNA3.1) GlyR subunits, plus mouse neuroligin 2A (pNice) and rat gephyrin (pCIS). In experiments described in **Figure 4**, we employed human α2 (pcDNA3.1), human α4 (pCIS), β2 (pcDNA3.1), and γ2L (pcDNA3.1) GABA_A_R subunits. Site-directed mutagenesis was performed using the QuikChange mutagenesis kit (Agilent Technologies) according to manufacturers’ instructions and the successful incorporation of mutations was confirmed by DNA sequencing.

### Patch Clamp Electrophysiology and Data Analysis

Standard patch-clamp electrophysiology equipment can be used, with the only specific requirement being a fluorescence microscope for identifying GFP fluorescent cells. Coverslips containing the co-cultured cells were placed gently into the recording chamber on the microscope stage and perfused continuously with an extracellular solution comprising (in mM): 140 NaCl, 5 KCl, 2 CaCl_2_, 1 MgCl_2_, 10 HEPES, and 10 D-glucose, adjusted to pH 7.4 with NaOH. Patch pipettes were filled with an intracellular solution containing (in mM): 145 CsCl, 2 CaCl_2_, 2 MgCl_2_, 10 HEPES, 10 EGTA, and 2 MgATP, adjusted to pH 7.4 with NaOH. HEK293 cell selection is largely a matter of trial and error. A good starting point is to select large, strongly fluorescent green cells that are closely surrounded by many neurons, especially small clumps of neurons. Cells with a textured (rather than smooth) appearance often yield abundant IPSCs.

The electrophysiological techniques may vary according to the experimental requirements. For example, if precise quantitation of rise times is required, it is extremely important that the filtering and digitisation rates are high and that pipette series resistance is low to avoid artefactually slowing down the event. In contrast, testing the effect of a drug on IPSC decay rate is less sensitive to filtering, and it may be necessary to use higher resistance pipettes to obtain a membrane seal that is stable enough to permit recordings that are long enough to apply and wash out the drug.

In all experiments described below, series resistance was compensated to 60% of maximum and was monitored throughout the recording. Spontaneous and action potential-evoked IPSCs in HEK293 cells were recorded at a holding potential -60 mV and currents were filtered at 4 kHz and sampled at 10 kHz. Only cells with a stable series resistance of <25 MΩ throughout the recording period were included in the analysis. Patch pipettes (4–8 MΩ resistance) were made from borosilicate glass (GC150F-7.5, Harvard Apparatus). Analyses of IPSC amplitude, 10–90% rise time, and decay time constant (single-exponential) were performed using Axograph × (Axograph Scientific). Single peak IPSCs with amplitudes of at least three times above the background noise were detected using a semi-automated sliding template. Each detected event was visually inspected and only well-separated IPSCs with no inflections in the rising or decay phases (suggestive of superimposed events) were included. The respective parameters from all selected events from a single cell were averaged and are presented as a single data point in **Figures 2–4**. The averages from multiple cells were then pooled to obtain group data. Statistical analysis and plotting were performed on group data with Prism 5 (GraphPad Software). All data are presented as mean ± SEM. One-way and two-way ANOVA were employed for multiple comparisons. For all tests, the number of asterisks corresponds to level of significance: ^∗^*p* < 0.05, ^∗∗^*p* < 0.01, ^∗∗∗^*p* < 0.001 and ^∗∗∗∗^*p* < 0.0001.

## Results

### Glycinergic IPSCs

While we found that some co-cultures exhibited spontaneous activity in almost all green fluorescent HEK293 cells, it was more typical to observe spontaneous glycinergic IPSCs in around 20% of cells. This success rate is adequate for most experiments.

It is important to establish that the spontaneous IPSCs produced by the co-culture synapses exhibit similar characteristics to those mediated by native synapses incorporating the same subunits. **Figure 2A** shows sample IPSC recordings from HEK293 cells expressing α1β GlyRs. An IPSC averaged from >200 events recorded from multiple cells is shown in **Figure 2B**. Mean amplitudes, 10–90% rise times and decay time constants are presented in **Figure 2C**. Frequency distributions of IPSC amplitudes, 10–90% rise times and decay time constants all exhibit monotonic distributions suggesting a single functional population of synapses (**Figure 2C**). In adult hypoglossal motor neurons, where the α1β GlyR isoform predominates (Lynch, 2009), the 10–90% rise times and decay time constants range between 0.6–1.8 and 4.9–7.7 ms, respectively (Singer et al., 1998; Graham et al., 2006; Hirzel et al., 2006; Muller et al., 2006). The mean decay time constant (9.3 ms) and the 10–90% rise time (1.9 ms) recorded in our co-culture synapses correspond well with these results.

We performed a similar analysis on α3β co-culture synapses and found the mean IPSC rise and decay times to be remarkably similar to those mediated by α1β GlyRs (**Figures 3A–C**). These parameters were also distributed monotonically, again suggesting a single population of synapses (**Figure 3C**). Although α3β-mediated IPSCs have yet to be recorded in isolation in native neurons, evidence to date suggests their rise and decay times are indistinguishable from those mediated by α1β GlyRs (Harvey et al., 2004). This fits well with the results from our engineered synapses.

The α1 GlyR subunit D80A and A52S mutations result in startle disease phenotypes in mice (Graham et al., 2006; Hirzel et al., 2006). We previously demonstrated that engineered synapses incorporating α1^D80A^β and α1^A52S^β GlyRs exhibited accelerated IPSC decay rates closely resembling those recorded in native synapses from mutant mice homozygous for these mutations (Zhang et al., 2014). This provides an important validation of our technique. In this study we sought to determine whether GlyRs may be located both synaptically and peri-synaptically in HEK293 cells by introducing mutations that dramatically enhanced the glycine sensitivity. We reasoned that if GlyRs are located peri-synaptically then enhancing their glycine sensitivity may render them susceptible to activation by synaptically released glycine, and if so this should be detectable as an additional slow rise time component (Wu et al., 2012). As noted above, α3^P185L^ results from a post-transcriptional RNA editing mechanism that is upregulated in (and is causative of) human temporal lobe epilepsy (Eichler et al., 2008, 2009). This mutation reduces the glycine EC_50_ from 70.9 to 7.4 μM (Legendre et al., 2009). We also investigated the α1^A288G^ mutation (which is not associated with a disease) because it reduces the glycine EC_50_ from 30.9 to 6.0 μM (Lynagh and Lynch, 2010).

As shown in **Figures 2A–C**, IPSCs mediated by α1^A288G^β GlyRs exhibited significantly slower rise times and decay time constants, although additional slow components were never observed on the rising phase of IPSCs. As with unmutated α1β GlyRs, these properties were monotonically distributed (**Figure 2C**) suggesting a single postsynaptic receptor population. Similarly, **Figures 3A–C** shows that IPSCs mediated by α3^P185L^β GlyRs also exhibited significantly slower rise times and decay time constants that were distributed monotonically. Thus, we were not able to unequivocally distinguish a putative peri-synaptic GlyR population in either case.

### GABAergic IPSCs

As with glycinergic IPSCs, we typically observed GABAergic IPSCs in around 20% of HEK293 cells. The rise times and decay time constants of IPSCs recorded from the dominant (α1β2γ2) synaptic subtype (1.2 and 4.0 ms, respectively) are in close accordance with those recorded from neurons known to predominantly express this subtype (Dixon et al., 2014). The co-culture system has revealed that other GABA_A_R subunit combinations can yield IPSCs with dramatically different rise and decay times (Wu et al., 2012; Dixon et al., 2014), although it is as yet unclear how these properties relate to those of the same isoforms when expressed in native neuronal synapses.

We have previously demonstrated that the effects of some clinically important drugs on co-culture GABAergic synaptic IPSCs are similar to those recorded at corresponding neuronal synapses. For example, 1 μM diazepam or 0.1 μM flunitrazepam significantly increased the decay time constants of IPSCs mediated by α2-containing GABA_A_Rs (Dixon et al., 2014) and 1 μM zolpidem or 1 μM eszopiclone increased IPSC magnitudes and decay time constants of IPSCs mediated by α1-containing GABA_A_Rs (Dixon et al., 2015). Here we extended this characterisation by performing a ‘reciprocal’ pharmacological comparison of α2β2γ2 and α4β2γ2 GABA_A_Rs, based the knowledge that α4-containing GABA_A_Rs are highly sensitive to ethanol and insensitive to benzodiazepines, whereas α2-containing GABA_A_Rs have the opposite profile (Knoflach et al., 1996; Wallner et al., 2006). As shown in **Figures 4A,B**, IPSCs mediated by recombinant α2β2γ2 and α4β2γ2 GABA_A_Rs exhibit identical amplitudes, 10–90% rise times and decay time constants. A physiologically relevant (10 mM) ethanol concentration significantly increased the IPSC decay time constant in α4β2γ2 GABA_A_Rs whereas 1 μM diazepam had no effect (**Figures 4C,D**). On the other hand, 1 μM diazepam significantly prolonged the IPSC decay time constant in α2β2γ2 GABA_A_Rs (Dixon et al., 2014), whereas even a very high (30 mM) ethanol concentration had no effect (**Figure 4D**).

## Discussion

### Applications of the Protocol

We have described protocols for reliably generating recombinant inhibitory synapses that incorporate defined GlyR or GABA_A_R isoforms of interest. These are suitable for investigating (1) the kinetics of IPSCs mediated by defined GABA_A_R or GlyR isoforms, (2) the effects of drugs on IPSCs mediated by defined GABA_A_R or GlyR isoforms, (3) the effect of posttranslational modifications (e.g., phosphorylation) and hereditary disease mutations on the formation and function of both types of synapses, and (4) synaptogenesis and synaptic clustering mechanisms in both types of synapses. We now expand on each of these points.

### IPSC Kinetics

Inhibitory post-synaptic currents mediated by different synaptic GABA_A_R or GlyR isoforms exhibit unique physiological and pharmacological profiles. It is useful to quantitate these properties because they may help in identifying the presence, or even the role, of a particular isoform in a particular neuron and also because accurate parameters provide key inputs into computational models of neuron or network function. Although studying recombinant receptors in standard heterologous expression systems such as HEK293 cells or *Xenopus* oocytes allows the electrophysiological properties of a single isoform to be studied in isolation, this approach is limited because the neurotransmitter must be applied artificially and it cannot mimic the fast (μs) dynamic neurotransmitter concentration profile that exists in the synaptic cleft.

### Investigating Drug Efficacy and Selectivity

The GABA_A_R is an established therapeutic target for clinical indications including epilepsy, anxiolysis, muscle spasms, sedation and anesthesia. GABA_A_R-targeted drugs currently in clinical use are not strongly subtype-selective and this can lead to dose-limiting side effects. For example, diazepam produces effective anxiolysis by positively modulating α2-containing GABA_A_Rs, although it also elicits the side effect of sedation by modulating α5-containing GABA_A_Rs (Trincavelli et al., 2012). Drugs specific for other isoforms are also being sought. For example, selective modulators of α5-containing GABA_A_Rs are being developed for a range of indications including stroke, cognitive impairment, and schizophrenia (Soh and Lynch, 2015). Although GlyRs are not currently targeted by clinically useful drugs, molecules that selectively enhance α3-containing GlyRs are considered promising as new generation treatments for chronic pain (Zeilhofer, 2005; Lynch and Callister, 2006). When evaluating new molecules as potential therapeutic lead compounds for synaptically localized receptors, it is important to test their potency, efficacy and subtype-selectivity under realistic synaptic activation conditions. The system we describe provides the most definitive means available of evaluating drug efficacy and selectivity at IPSCs mediated by defined receptor isoforms.

### Investigating Disease Mutations

Engineered synapses have yet to be used to study disease-causing GABA_A_R mutations or modifications, and hence, the method has not realized its full potential as a model system for understanding the molecular pathology of neurological disorders. Mutations in GABA_A_R α1 and γ2 subunits have long been associated with genetic epilepsy syndromes (Macdonald et al., 2010). Thus far, the function and pharmacology of epilepsy-causing mutant GABA_A_Rs have only been investigated using whole-cell recordings of steady-state GABA-activated currents in heterologous expression systems. The differing approaches that have been used to analyze the effects of these mutations have lead to controversy, particularly in the case of the γ2*^R43Q^* mutation (Petrou and Reid, 2012). Moreover, of all the identified epilepsy-causing mutant GABA_A_Rs that exhibit partial or full expression at the cell membrane, there is an animal knock-in model of only one (Petrou and Reid, 2012). Transgenic animal models are aﬄicted by compensatory mechanisms that can obfuscate data, especially those involving ion channel genetic manipulations that affect GABAergic transmission (Harris et al., 2011). Because co-culture α1β2γ2 GABA_A_R synapses successfully recapitulate the kinetics of neuronal IPSCs (Dixon et al., 2014), they may provide a promising means of investigating the synaptic signaling defects induced by hereditary epilepsy mutations to α1 and γ2 subunits.

Glycinergic co-culture synapses incorporating α1^D80Aβ^ and α1^A52Sβ^ GlyRs have been shown to exhibit accelerated IPSC decay rates that strongly resemble those recorded in native synapses from mutant mice homozygous for the same mutations (Zhang et al., 2014). This suggests that the co-culture system should be useful for modeling the effects of hyperekplexia mutations to α1 and β subunits (Bode and Lynch, 2014) and autism mutations to α2 subunits (Pilorge et al., 2015). Here we investigated the effect of the α3^P185L^ mutation that is associated with temporal lobe epilepsy. The mutation resulted in slowing of the IPSC decay rate (**Figure 3**). However, glycinergic synapses are absent in the temporal lobe and it is thought that the affected receptors are located presynaptically at glutamatergic synapses. In this case, our results suggest the mutant GlyR would remain open for longer during each synaptic event, thus potentiating an excitatory Cl flux leading to enhanced excitatory neurotransmission that could underlie the disorder (Eichler et al., 2008, 2009; Legendre et al., 2009).

### Synaptogenesis and Synaptic Clustering Mechanisms

Co-culture synapses have been used extensively to probe the roles of synaptic adhesion molecules in the formation of glutamatergic or GABAergic synapses (Scheiffele et al., 2000; Biederer et al., 2002; Dean et al., 2003; Graf et al., 2004; Sara et al., 2005; Kim et al., 2006; Dong et al., 2007; Fuchs et al., 2013) or to investigate the impact of disease-causing neuroligin mutations on GABAergic synaptogenesis (Chubykin et al., 2005; Sun et al., 2011). The strengths and weaknesses of co-cultures in this respect have recently been reviewed (Fuchs et al., 2013).

## Conclusion

As the presynaptic terminals of our engineered synapses are provided by real neurons, their function is likely to resemble those of synapses *in vivo*. Indeed, serial electron microscopic reconstructions of GABAergic terminals onto HEK293 cells have confirmed that their ultrastructures are similar to those of native neurons (Fuchs et al., 2013). However, the postsynaptic specializations are less likely to resemble those of neurons given that HEK293 cells do not endogenously express all necessary postsynaptic clustering proteins at appropriate levels for synaptogenesis. In addition, some proteins that they do express may lack neuron-specific post-translational modifications required for correct synaptic function. These factors could ultimately alter the geometry of the synaptic cleft and the postsynaptic receptor clustering density, leading to non-physiological changes in the neurotransmitter concentration profile that could affect IPSC kinetics. This uncertainty is the main limitation of the technique. We have addressed this as far as possible by comparing the properties of engineered synapses with those of real synapses in cases where we can be reasonably sure about the synaptic subunit composition.

However, due to their non-physiological status, engineered synapses also offer opportunities to investigate new clustering mechanisms. If, for example, substitution of a particular pLGIC subunit results in a drastic, unexpected slowing of the IPSC rise time, it is possible that synaptic receptors have been de-clustered in a manner that may not occur in a neuron. This could in turn lead to the identification of novel clustering molecules and mechanisms. HEK293 cells are ideal for investigating such questions: they do not express all proteins necessary for synaptogenesis, but they do provide a high efficiency of transfection, faithful protein translation and a small, electronically compact shape appropriate for accurate quantitation of IPSC rise and decay times (Thomas and Smart, 2005).

## Author Contributions

CD, YZ, and JL conceived the project and developed the protocols; CD and YZ performed experiments and analyzed the data; and CD, YZ, and JL wrote the manuscript.

## Conflict of Interest Statement

The authors declare that the research was conducted in the absence of any commercial or financial relationships that could be construed as a potential conflict of interest.
