# Supplementary material for: Generation of Functional Inhibitory Synapses Incorporating Defined Combinations of GABA(A) or Glycine Receptor Subunits
Source: Front Mol Neurosci. 2015 Dec 23;8:80. doi: 10.3389/fnmol.2015.00080 (PMC4688394; doi:10.3389/fnmol.2015.00080)
Supplement: Supplementary file 1 [file Presentation_1.PDF]

Supplementary Information

Protocol for generating functional inhibitory synapses between neurons and HEK293 cells expressing defined combinations of GABA(A) or glycine receptor subunits

REAGENTS

- Timed-pregnant rats at E15 (for spinal cord) or E18 (for cerebral cortex).
- Trypsin 0.05 % in EDTA with phenol red (Gibco 25300-054, Life Technologies)
- Divalent cation free Phosphate-buffered saline solution (PBS)
- Poly-D-lysine (P6407-5MG, Sigma-Aldrich)
- Fetal Bovine Serum (FBS, sterile filtered, Batch number 28301107, Moregate Biotech)
- Dulbecco's modified Eagle's medium (DMEM; Gibco 11995-065, Life Technologies)
- Neurobasal media (Gibco 21103-049, Life Technologies)
- B27 (Gibco 17504-44, Life Technologies)
- Glutamax (Gibco 35050-61, Life Technologies)
- HEK293 cells. We usually use the HEK293 variant AD-293 (240085, Agilent), but have also successfully used Cos9 cells and HEK293T cells.
- Plasmids for: Neuroligin 2A (plasmid 15259, Addgene), green fluorescent protein (GFP), and receptor subunits of interest.
- Hank's Balanced Salt Solution (HBSS; Gibco 14025, Life Technologies)
- Calcium-magnesium free HBSS (CMF-HBSS; H9394, Sigma-Aldrich)
- Trypan blue 0.4 % solution (Fluka 93595 Sigma-Aldrich)
- DNase I (10104159001, Roche Applied Science)
- $CaCl_2$, 250 mM in milliQ water, sterile filtered
- N,N-bis(2-hydroxyethyl)-2-aminoethane- sulfonic acid (BES) buffered saline (BBS; 14280, Sigma-Aldrich)

EQUIPMENT

- 4-well plates (e.g. 176740, Nunclon). We find that multi-well plates are less vulnerable to infection than 35 mm dishes.
- Circular glass coverslips (12 mm diameter, Menzel #1.5, Thermo Fisher Scientific)
- Fine forceps, curved forceps and dissection scissors for embryonic dissection.

- Dissection tools for adult rat
- CO_2 supply and chamber for euthanasia
- 70 % ethanol in spray bottle
- Lab wipes
- Tube rack
- Ice bed
- 100 mm petri dishes
- 50 ml tubes
- 15 ml tubes
- 1.5 ml microcentrifuge tubes
- Pipettes and tips
- Glass pasteur pipettes
- Cigarette lighter
- Rubber bulb for pasteur pipette
- Haemocytometer
- 35 mm dishes for HEK293 cells
- T75 flasks
- Dissecting microscope with light
- Cell culture cabinet (Class II biological safety cabinet or laminar flow hood)
- Centrifuge with rotor for 15 ml tubes
- Bottle/tube roller
- Humidified cell culture incubator at 37 °C, with 5 % CO_2
- Humidified cell culture incubator at 37 °C, with 3 % CO_2 (If unavailable, transfection will still be partly effective at 5 % CO_2)
- A standard patch-clamp electrophysiology rig incorporating either an upright or an inverted fluorescence microscope.

EQUIPMENT SETUP

- Clean glass coverslips: Clean coverslips in bulk by rinsing for 10 min in concentrated hydrochloric or nitric acid.

  CAUTION: When handling acid wear gloves, safety glasses and a lab gown and work in a fume hood. Remove the acid and wash coverslips in running distilled water until the pH returns to neutral (usually 1 – 2 h). Pour off water and store coverslips in a sealed glass container under 100 % ethanol.

REAGENT SETUP

All reagent setup steps should be performed in a cell culture cabinet.

- DMEM-FBS media: DMEM plus 10 % FBS.
- Aliquot B27 in 1 ml tubes and freeze for up to 4 months. Once thawed use aliquots within 1 week.
- Neuron media: In a 50 ml tube combine 1 ml B27, 0.5 ml Glutamax supplement and top up to 50 ml with Neurobasal medium. Loosen the lid and equilibrate to 37 °C and 5 % CO_2 in the cell incubator before use.
- Dilute DNAse I to 10 mg/mL in CMF-HBSS, and stored frozen 1 mL aliquots for up to 6 months.
- Coated coverslips: Make poly-D-lysine stock solution by adding 5 ml cell-culture water to 5 mg of poly-D-lysine, and freeze as 0.5 ml aliquots for up to 6 months. Remove ~40 cleaned coverslips from the ethanol (see equipment setup) and, in a 50 mL tube, rinse with cell culture water. Dilute 1 aliquot of poly-D-lysine stock to 5 ml, and incubate the coverslips on a tube roller for at least 1 h at room temperature. Pour off poly-D-lysine solution and use forceps to spread coverslips out to dry on sterile lab tissue in the cell culture cabinet. **PAUSE POINT:** Poly-D-lysine solution can be refrigerated and re-used within 1 wk. Coated coverslips are best used within 1 wk, but can be stored in a dry sterile tube at room temperature for 1 month.

PROCEDURE

Primary culture

1. Coat coverslips and place one coverslip in each well of 16 x 4-well plates.
2. Outside of the biological safety cabinet, euthanize the pregnant rat with CO_2, open the abdomen and quickly recover the embryos into tubes of chilled CMF-HBSS. If necessary, the embryos can remain on ice for 2-3 h prior to the rest of the procedure.
   CAUTION: Euthanasia of animals must be carried out in accordance with national and institutional guidelines.
3. Steps 3–16 below are carried out within a cell culture cabinet using sterile techniques. Begin with option A for cortical cultures or option B for spinal cord cultures.

A) Isolating cortical neurons (for GABAergic synapses)

i) Remove the heads from 3 embryos and rinse in a 100 mm petri dish containing chilled CMF-HBSS. Fixing each head with straight forceps, use curved forceps to

peel back the skin and skull. Pinch off the top 1 mm of cortical tissue, and place in a fresh dish of CMF-HBSS.

ii) Use fine forceps to peel off the meninges, by grasping blood vessels at the edge of the tissue and teasing them away.

CRITICAL STEP: Failure to remove the meninges will increase glia cell numbers in the culture, decreasing the health and lifespan of the neurons.

iii) Use the flame from a cigarette lighter to lightly polish the tip of a sterile glass Pasteur pipette. Suck up a little fresh CMF-HBSS and gently rinse the cortices, then suck them up and place in a sterile 15 ml tube.

B) Isolating spinal cord neurons (for glycinergic synapses)

i) Remove the head and tail of each E15 embryo using forceps, and transfer it to a 100 mm petri dish filled with ice-cold CMF-HBSS under a dissecting microscope.

CRITICAL STEP: The dissection procedure should be performed on ice.

ii) Place the embryo in the prone position and stabilize the embryo with straight forceps. Slide the other pair of forceps to cut the skin and meninges along midline to open the spinal cord.

iii) Poke the tissue closed to the dorsal lip of the spinal cord with closed forceps. Open the forceps to tear dorsal tissue away. Repeat this operation to detach surrounding tissue from both sides of the spinal cord.

iv) Pin the wider, proximal end of spinal cord with forceps and peel off meninges from the spinal cord.

CRITICAL STEP: Slowly and evenly peel off the meninges and avoid breakage of the spinal cord or the meninges. Failure to remove the meninges will increase the numbers of glial cells in the culture.

v) Use fire-polished glass Pasteur pipette to transfer the isolated dorsal strips to a fresh petri dish containing ice-cold CMF-HBSS.

vi) Collect 7 intact dorsal strips into a 15 ml tube containing 4 ml of 0.05 % trypsin and 1 ml DNase I.

vii) Incubate at 37 ^{o}C in the water bath for 20 min with intermittent agitation.

viii) Stop the digestion by adding 5 ml of trypsin inhibitor (or DMEM/FBS media), and centrifuge at 100 g for 3 min at room temperature.

ix) Discard the supernatant. Wash the tissue pellet by adding 2 ml CMF-HBSS.

4. Use a fire-polished glass pipette to triturate the tissue, gently pipetting up and down 5 to 10 times, taking care to avoid bubbles. Small chunks of tissue should still remain, but the media will become cloudy as cells are released.

   CRITICAL STEP: Pipetting speed needs to be low and the inside diameter of the glass pipette tip is critical. If it is too large it will not dissociate the cells effectively, and if it is too small it will increase cell damage. The optimal size depends on the size of tissue chunks. One solution is to sequentially triturate with three pipettes with decreasing diameters.

5. Allow tissue pieces to settle for 1 min. Then aspirate the supernatant into a fresh 15 ml tube and centrifuge for 3 min at 100 g at room temperature.

   CRITICAL STEP: Be careful not to aspirate the settled debris.

6. Remove supernatant. Gently resuspend pellet in 1 ml of DMEM-FBS.
7. In a 1.5 ml tube, mix 10 µl trypan blue with 10 µl of cell suspension. Load 10 µl into the haemocytometer and count the viable (non-blue) cells, remembering to multiply the final count by 2 to account for the trypan blue dilution. Typically, cortices from 3 embryos or spinal cords from 7 embryos will result in 200-500 cells per 0.1 µl.
8. Dilute cells in DMEM-FBS so that 80 – 100 µl can be plated on each coverslip of 8 dishes, resulting in 80,000 cells per coverslip. Dilute the remaining cells 2-fold, and plate the remaining 8 dishes at 40,000 cells per coverslip.
9. Incubate overnight, and then change media by aspirating the DMEM-FBS and replacing with 500 µl neuron media. Return dishes to the incubator
10. After 1 week in vitro, perform a half media change by removing 250 µl media and topping up with a further 500 µl of fresh neuron media. This is the final feed and will allow the cells to remain healthy for up to 6 weeks.

Preparation of HEK293 cells

11. Trypsinize HEK293 cells in a T75 flask and plate in DMEM-FBS onto 35 mm culture dishes (suggested starting density: 5000 cells per dish). Incubate overnight at 5 % CO_2.
12. To transfect, combine 0.5 – 3 µg total plasmid DNA, with 100 µl of 250 mM $CaCl_2$, then add 100 µl BBS. Vortex and incubate for 10 min at room temperature, then add dropwise to one 35 mm dish of HEK293 cells. When expressing GlyRs, the total plasmid DNA should comprise: 200 ng neuroligin 2A, 200 ng gephyrin, 100 ng GFP, with the remainder comprising GlyR subunit DNA that varies according to the number of subunits and the ratio of subunit DNA required. For example, when transfecting GlyR α subunits as homomers, add 0.5 µg DNA. When transfecting α and β GlyR subunits in 1:10 or 1:50 ratios add a total

of 2 μg DNA. When expressing α1β2γ2 $GABA_ARs$, the α1, β2, γ2, GFP and neuroligin 2A DNAs should be transfected in a 1:1:4:1: ratio with a combined total of 0.5 μg DNA.

13. Incubate for 5 to 20 h in a 3% CO_2 incubator.
14. Thoroughly rinse each plate with PBS, then incubate in 0.5 ml trypsin until most cells detach (typically 3 min). Quench with 2 ml DMEM-FBS, and remove cell suspension into 15 ml tube. Centrifuge at 100 g for 3 min at room temperature.
    Discard supernatant.
15. Take mature neuron cultures from the incubator and remove around 50 μl from each dish to combine with the HEK293 cell pellet. One 35 mm dish of HEK293 cells can be used to seed about 4 coverslips of neurons.
    CRITICAL STEP Thoroughly resuspend the HEK293 cell pellet in neuron media so that all cells are completely isolated. Well isolated cells are essential for allowing the efficient formation of synaptic contacts and to facilitate electrophysiological recording from identified cells.
16. Return the co-cultures to the incubator overnight to allow synapses to form. Cultures can then be used for 2 – 3 d.

Electrophysiological analysis of spontaneous activity

17. Using fine forceps, place a coverslip gently into an electrophysiological recording bath on the stage of the fluorescence microscope that forms part of the patch-clamp electrophysiology rig. The cells should be perfused continuously with an extracellular solution comprising (in mM): 140 NaCl, 5 KCl, 2 $CaCl_2$, 1 $MgCl_2$, 10 HEPES, and 10 D-glucose, adjusted to pH 7.4 with NaOH. Depending on the experiment, pharmacological agents may be added or the ionic composition may be varied. Because drugs that affect IPSCs may also change the rate of spontaneous activity in the neurons, it is advisable to apply drugs directly to the patch-clamped cell, and expose the rest of the coverslip to control saline as much as possible. We achieve this using a small array of plastic tubes glued together, with control solution flowing through all but the central tube.
18. Patch pipettes (4−8 MΩ resistance), made from borosilicate glass (GC150F-7.5, Harvard apparatus), should be filled with an intracellular solution containing (in mM): 145 CsCl, 2 $CaCl_2$, 2 $MgCl_2$, 10 HEPES, 10 EGTA and 2 MgATP, adjusted to pH 7.4 with NaOH. The ionic composition can be varied according to experimental requirements. Heat polishing the pipette tips can help with seal formation, but be aware of the effect of increased series resistance on rapid events (see next step).

CRITICAL STEP: As IPSCs exhibit rise times in the microsecond range, it is important to filter and digitise currents at a high rate (at least 4 and 10 kHz, respectively) and to apply the maximum possible series resistance compensation.

19. Choosing a cell for electrophysiological recording is a matter of trial and error. A good starting point is to select large, strongly-fluorescent green cells that are closely surrounded by many neurons, especially clumps of neurons. HEK293 cells with a textured (rather than smooth) appearance often yield abundant IPSCs. A good rule of thumb is to target cells that look similar to those that have previously yielded successful recordings.
20. Voltage clamp cells at -70 mV to -50 mV to record spontaneous events for several minutes, or until more than 100 putative events have been collected.
21. The coverslip should be viable for 1 − 2 h. Discard it sooner if cell viability starts to appear degraded. Unused coverslips in 4-well plates should be returned to the incubator as soon as possible to maintain the pH of the medium.
22. Analyses of IPSC amplitudes, 10 − 90% rise times, and decay time constants can performed using a range of analysis software packages including Axograph X (Axograph Scientific), Minianalysis (Synaptosoft) and pClamp10 (Axon Instruments, Molecular Devices).

TIMING

See Figure 1 for an overview of the procedure. Set-up and culturing of primary neurons takes 2 to 3 h (the dissection for spinal cord cultures is longer). Then the neurons must be cultured until maturity, at least 3 wk for cortical neurons and 1 – 1.5 wk for spinal cord neurons. Preparing the HEK293 cells and adding them to the neurons takes about 2 h spread across 3 d. Once HEK293 cells have been added to the neurons, the synapses form within 24 h and at this point the electrophysiological experiments can be commenced.

TROUBLESHOOTING

- Not enough neurons for plating: If there are not enough cells to fill the intended plates, trituration may have been too vigorous (more then 50 % of cells trypan positive) or too gentle (few trypan positive cells and few negative) Experiment with trituration of different strengths. As noted above, the tip size of the flame-polished glass pipette is crucial: a very small opening will damage more cells, but a large opening will not dissociate the cells effectively.
- Massive cell death after seeding: Check the incubator CO_2 level, temperature, and water levels; check the temperature of the trypsin incubation; make fresh neuron media or coverslip coating solution.

- Difficulty forming seals on HEK293 cells: Co-cultured cells are often more difficult to record from than HEK293 cells that have been cultured in isolation. Fire polishing the patch pipette tip can help to form high resistance seals, but be aware of increasing the series resistance.
- Unhealthy neuron cultures leading to little spontaneous activity: Too many glia present in the final cultures (take more care to remove the meninges), or plating density too high or too low.
- Non-transfected HEK293 cells: Adjust DNA ratios, or try fresh BBS. Alternately, other methods of transfecting HEK293 cells could also be applied.
- No detectable spontaneous IPSCs in the HEK293 cells: Check that the cells are expressing functional receptors by directly applying the appropriate neurotransmitter agonist. If there is no current, trouble-shoot the transfection. If agonist-evoked currents are present, check that the neurons exhibit spontaneous spiking activity by recording from a few. If they are not active, the neuronal cultures may be too young or too old, the neuron density may be too high or too low (leading to cell death) or excess glial growth may be impairing neuronal activity. Check that neuroligin is being successfully expressed using immunofluorescent labelling for the HA tag (as shown in Figure 1, inset). If all of these things seem normal but brightly fluorescent HEK293 cells still do not have currents, consider patching cells with different appearances, including those with low fluorescence. We find that large or brightly fluorescent HEK293 cells next to clumps of neurons have the highest probability of showing synaptic activity.
